# “Give me some space”: exploring youth to parent aggression and violence

**DOI:** 10.1007/s10896-017-9928-1

**Published:** 2017-10-02

**Authors:** Lynne Gabriel, Zahra Tizro, Hazel James, Jane Cronin-Davis, Tanya Beetham, Alice Corbally, Emily Lopez-Moreno, Sarah Hill

**Affiliations:** 1 York St John University, York, England YO31 7EX UK; 2 UK Higher Education Academy, Science Park, Heslington, York, UK; 3 St George’s University of London / Kingston University, Cranmer Terrace, London, SW17 0RE UK; 4 Sheffield University, Sheffield, UK; 5 Independent Domestic Abuse Services (IDAS), 39 Blossom Street, York, UK

**Keywords:** domestic violence, aggression, youth, family dynamics, trauma

## Abstract

A small scale qualitative project, undertaken by an interdisciplinary domestic violence research group involving academic researchers and research assistants, with colleagues from Independent Domestic Abuse Services (IDAS), investigated youth aggression and violence against parents. Following the literature review, data was generated through several research conversations with young people (*n* = 2), through semi-structured interviews with mothers (*n* = 3) and practitioners (*n* = 5), and through a practitioner focus group (*n* = 8). Thematic analysis and triangulation of the data from parents, practitioners and young people, elicited interconnected and complex overarching themes. Young people could be both victim and perpetrator. The witnessing or experiencing of domestic aggression and violence raised the concept of ‘bystander children’. The impact of young people experiencing familial violence was underestimated by parents. For practitioners, the effects of working with domestic violence was shown to be significant - both positively and negatively.

## Introduction

Domestic violence and abuse touches all ages, cultures, and classes. According to an NSPCC study (2011), 12% of under 11 s, 18% aged 11–17 and 24% of those aged 18–42 have been exposed to domestic abuse between adults in their homes during childhood. Such exposure can have a negative impact upon young peoples’ health and wellbeing (Jofre-Bonet et al. 2016). Domestic violence is a matter of serious societal concern and Government attention, as well as a challenging issue for domestic violence agencies, frontline health or social care workers and statutory services. Identifying what prompts young people from domestic violence settings to progress to become perpetrators of violence themselves is under-researched (NICE 2014). Engaging in exploratory research conversations with young people, their parents and the practitioners who work with them, to better understand victim and perpetrator behavior, offers the possibility of deeper understanding, effective interventions and the prevention of future violence.

This small-scale qualitative study explored youth to parent aggression and violence and was a joint project with Independent Domestic Abuse Services (IDAS, a non-statutory organization working with victims and perpetrators of domestic violence and abuse). When we refer to youth, we mean young people aged 11–17. When using the term ‘practitioner’, these are professionals who work directly with victims and perpetrators of interpersonal or domestic abuse and violence, including statutory services and voluntary sector workers in support and guidance roles. We adopt the term *domestic violence and abuse* (DVA) to refer to familial and domestic violence (Howarth et al. 2015). In defining youth to parent aggression, we regard it as “…a pattern of behavior that uses verbal, financial, physical or emotional means to practice power and exert control over a parent…the exercise of control is usually evidenced by the parent’s inability to ‘do anything about it’” (Holt 2013, p. 1).

## Literature Review

Young people who use violence at home tend to perceive their parents as weak and ineffective and see themselves as lacking power (Routt and Anderson 2011). Typically, they are impulsive and emotionally driven and intentionally seek to hurt the parent (Routt and Anderson 2011). Violence can relate to a young person’s belief that their entitlement outweighs responsibilities for acceptable behavior (McKenna et al. 2010). Additionally, as children get older, abuse can progressively worsen with the increasing age or physicality of the child (Cottrell 2001; McKenna et al. 2010; Walsh et al. 2011).

Youth to parent violence often goes unreported (Holt 2011). Parents may deny the abuse or mitigate its severity (Walsh and Krienert 2007). Furthermore, it may be shaming for parents to disclose they have an abusive child, with self-doubt and ruminations such as *what does my child being abusive towards me say about me as a parent* undermining a parent’s ability to speak out. Bobic (2004) reports on this emotional impact, noting that stigma, guilt and shame are often influencing factors as to why parents do not report abuse. A parent’s own experience of childhood abuse or violence can undermine their adult mental health and their ability to provide appropriate and consistent parenting, further inhibiting disclosures (Holt 2011).

There are thought to be connections between witnessing domestic violence and the occurrence of youth to parent violence (Calvete et al. 2015; Edenborough et al. 2008; Ehrensaft et al. 2003; Routt and Anderson 2011; Widem and Wilson 2015). The severity of the violence could be indicative of how traumatic the original DVA situation was for the young person (Calvete et al. 2015; Holt 2013; Routt and Anderson 2011). Worryingly, those with a history of childhood abuse and neglect are three times more likely to go on to perpetrate physical or sexual abuse in adulthood (Milaniak and Widon 2015).

Developmental issues arising from exposure to domestic violence include relationship problems and aggression in parental and sibling relationships (Calvete et al. 2015; Hague 2012; Hester et al. 2007). Wolfe et al.’s (2003) meta-analysis examined 41 studies on the effects of exposure to DV and found that 40 indicated impact on social, emotional, behavioral, and cognitive functioning, with effects potentially lasting into adulthood (Hague 2012).

To complement a developmentally influenced approach to DVA, evolutionary and biological positions suggest the value of viewing aggression and violence through a conceptual framework that recognizes both genetic and sociological influences (Ferguson and Beaver, 2009). Similarly, Strauss (2008) calls for DVA to be examined through a multi-causal model. Several studies have highlighted the social construction of gender and gendered roles, indicating social learning and social control are contributory factors, with more boys than girls hitting their caregivers (Kennedy et al. 2010; Langhinichsen-Rohling et al. 1995). Langhinichsen-Rohling et al. (1995) found that girls who witness parental violence are less likely to become violent. Girls are thought to be more likely to internalise distress or trauma, whereas boys tend to externalize it; hence the suggestion that girls tend towards verbal abuse and boys towards physical aggression (Cottrell 2001; Pagani et al. 2009). Conversely, Walsh and Krienert (2007) found that girls have a higher prevalence of child to parent violence (CPV). Coogan (2011) found that as adolescent boys grow older, they are more likely to display violent and aggressive behavior towards their fathers, making the father a more likely target for CPV than the mother. Whilst there are diverse findings on the gender of the young perpetrator, most literature (for example, Cornell and Gelles 1982; Holt 2013; McKenna et al. 2010; Routt and Anderson 2011) identifies mothers as the commonest victim, with verbal abuse a typical medium for aggressors (McKenna et al. 2010).

In relation to parental influence Calvete et al. (2015) suggest that parenting styles have a significant impact in domestic violence situations, with children of parents who are overly permissive or lacking in warmth more likely to adopt maladaptive or aggressive coping tactics. Other studies have linked lack of parental authority with youth to parent abuse (Holt 2013; Ulman and Strauss 2003), whilst family life, socio-economic disadvantage, and compounding factors such as substance abuse, have also been identified as significant risk factors (McKenna et al. (2010). A recent study implies that mild physical reprimanding of children (mild spanking) does not adversely affect a child (Ferguson 2013), implying non-violent disciplining of a child is regarded differently from more severe punishment which constitutes domestic violence (Baumrind et al. 2002; Larzelere et al. 2010).

A Government focus upon troubled families (as in the UK), in particular those where aggression and violence feature, has operated largely through statutory and criminal justice channels and has been aimed at minimizing offending behavior. However, concerns have been raised in the US about this approach, with research evidence indicating that such programs fall short and provide inadequate support and interventions (Hamby et al. 2015)*.*

A range of complex and diverse factors appear to influence youth to parent violence. That said, in-depth qualitative studies that focus upon the lived experiences of young people and parents exposed to domestic violence are lacking; hence the present project, with its aim of exploring what triggers youth to parent aggression, from the perspective of the young person, parent and practitioner.

## Project Methodology

The project investigated ‘*What triggers young people to engage in aggression and violence with parents?’* through a critically reflexive, small-scale in-depth qualitative design (Ramazanoglu and Holland 2002; Tizro 2012). The research team worked in collaboration with IDAS to develop the aims of the project, and to design and conduct the study. A total of 18 participants were recruited using purposive and opportunistic sampling tactics. Data were generated through semi-structured interviews with parents (*n* = 3) and with practitioners (*n* = 5); a practitioner focus group (*n* = 8), and extended research conversations with young people (*n* = 2). Practitioner participants included police, social work, youth work, probation, and case workers from voluntary agencies. Interviews were undertaken with practitioner participants who were not able to attend a focus group meeting. The practitioner participant interviews and focus group took place on University premises. Parent and youth participants were recruited through IDAS. Their interviews took place in homes, school settings, or parental business settings. The parent and youth participants had previously taken part in a Respect program, delivered by IDAS. The program aims to change offending or unhelpful behaviors and elicit positive relational styles. The research conversations and interviews ranged between 30 and 90 min in length and enabled in-depth participant exploration of sensitive matters. The focus group lasted 90 min and was deemed to be an efficient way to capture a range of practitioner perspectives (Seale 1998). There was no overlap of participants across the focus group, research conversations and interviews.

The project research team included academic researchers (ARs, *n* = 4) based in a university and research assistants (RAs, *n* = 3) based in the same university. The ARs interviewed the mothers, facilitated the focus group and interviewed practitioners, whilst a senior research assistant worked with the ARs and IDAS to devise content and structure for research conversations with young people (Gabriel et al. 2017). Throughout, ethical considerations, researcher actions and project decision-making, were reflexively managed (Gabriel 2009, 2016; Tizro 2012) to help deal with the complex, sensitive and emotive topic (Downes et al. 2013). Critical reflexivity was central (Gabriel et al., 2017) and involved debriefing sessions with all the interviewers by the senior staff involved with the project, the use of reflexive diaries and joint interrogation of the data generated. All interview and focus group data were subjected to iterative cycles of thematic analysis and to peer review of coding, to elicit principle themes and sub-themes by the ARs (Gregory et al. 2012; Hayes 2000).

Ethical approval was granted by the Faculty Research Ethics Committee. Age appropriate project information was made available to the young participants and informed consent for their participation was sought from their parents. All adult participants and parents were given comprehensive details of the research project, to facilitate informed assent and consent processes. A steering group, comprising external stakeholders, the student RAs and the project researchers, supported the governance and quality assurance aspects of the research.

## Findings

A process of inductive thematic data analyses (Gregory et al. 2012; Hayes 2000) elicited core themes, which were triangulated across the parent, child and practitioner data sets and denoted complex, multi-faceted and interconnecting concepts. The nine core themes were: *both victim and perpetrator, relational power, boundaries, gender issues, social media, coping tactics, parental paralysis, wounded healers* and *bystanders.* Each theme is outlined below and pseudonyms are used when providing verbatim extracts from the dataset.

### Both victim and perpetrator

The young person and the parent could concurrently or sequentially occupy a victim or perpetrator position. The mothers and their children were direct victims of, or witnesses to, violence. Witnessing and being subjected to physical and psychological abuse impacted the subsequently aggressive or violent child in multiple ways. One mother, recognizing the damage caused to her aggressor daughter, Evie, by her former violent partner, commented that:

“When she is in touch with him she’s just in emotional turmoil. So much so, she was so stressed that she was getting facial tics. And she was, well lots and lots of weird things were happening. She started eating her fingers”.

The young person here was both a victim and an aggressor, who had displayed conflicting behaviors with her mother; at once seeking and repelling aggressive contact.

Another mother talked about how her aggressor son ‘…kept all of the anger inside and he took it out on me…” She described how, unbeknown to her, when the boys had access visits to their father he subjected them to cruelty and violence…

“…one particular day his dad punched [him] and [he] ran out the house … and they hid and they didn’t dare go back until his partner was back in the house….he left them on the top of a mountain in a snow storm …he left [him] in a hotel ….if only I’d known then what I know now”.

### Relational power

The notion of relational power-plays and the exercise of control was common across all participant groups. Whilst the young person, parent and practitioner themes could be differentiated, core aspects were evident. For example, one practitioner noted how power and control could be age and stage related. He commented on the developmental, relational and physiological challenges for young people displaying aggression and violence in the family

“…adolescents are in a transient stage…it’s this pattern of behavior where it’s power and control ….it is a consequence of not having proper boundaries put in when they’re younger, so what you’re seeing is continuation of a temper tantrum, but the thing is, they’re 14 when they’re doing it…”

According to Evie’s mother, “…she likes to be right and if she’s vulnerable then she’s lost that power”. This dynamic was often a precursor to the daughter’s aggression, concurrent with a mother who did not know how best to respond or mediate the situation. Equally, Evie was unclear about how to behave in the child-parent relationship and this was often manifest as aggression towards her mother. She found it difficult to mediate between the powerful paradoxical emotions of love and hate.

Lack of positive role models for children and young people was common across the practitioner, parent and child data sets. Reporting on their work with a female co-worker, one male practitioner noted:

“…our working relationship is a good role model for these young perpetrators and some ….can’t believe how much respect I’ve got for [her] and how much [she’s] got for me and they, they, you know, we’re good friends out of work and they’re sort of like well ‘why you being nice to her?’”

Role confusion was most evident once the offending parent had left the domestic setting. This could play out through the young person taking on the aggressor role in the household, or feeling confused about who to follow or trust. Positive behavior change needed a facilitative medium, as in the case of George, whose savior was a male case worker from IDAS. They enabled him, through a child-centered and creative approach, to talk through his experiences and identify helpful behaviors and coping tactics, along with positive ways forward in his relationship with his mother.

Role reversal was common. George’s mother noted “I don’t like to say the word ‘victim’, I did what he said and you know, I, the roles were reversed almost…” and she felt unable to disclose this to anyone. Positive changes in the child-parent dynamic were only made possible through third party interventions to alter the status quo. Here, Respect’s youth perpetrator program, as delivered by IDAS, had a significant positive impact upon all of the mothers.

For practitioners, role conflict occurred in several contexts. In particular, practitioners referred to funding cuts and related reduction in staffing. They felt deskilled in their roles in the face of seemingly intractable situations, lack of connectivity across agencies and the lack of funding for their organizations or work program. The work could prompt their own trauma through confronting human violence and vulnerability in their day to day work.

### Boundaries

Parents and practitioners described relational and situational boundary issues. For all three mothers, managing appropriate boundaries with their children had been especially challenging until they entered the Respect program at IDAS. Each noted the positive impact of the program workers and the good working relationships both they and their children experienced with the practitioners. Learning how to manage boundaries was an important outcome for all three mothers.

One mother conveyed her ambivalence and ambiguity around boundaries in the mother-daughter relationship “…I’m not very good with the rules and I’m not very consistent and I do things on the spur of the moment ..” and “I just find it a bit strange…children are supposed to push boundaries…”. The mother’s turmoil was palpable. For Evie, consuming and paradoxical emotions made it difficult to understand ‘acceptable’ boundaries.

This was also the case with George, who conveyed intense fear, anxiety and frustration in relation to appropriate boundaries for contact with his abusive father. Violations of personal space and relationship boundaries were evident in all three families, with concurrent difficulties around self-agency, trust and capacity to be in a ‘healthy’ child-parent relationship.

### Gender issues

Prompted by powerful emotions such as fear or anxiety, a young person may defer to the perpetrator parent; usually the male parent or caregiver. Practitioners reported this was often the case with male perpetrators, whilst the young people reported instances of a violent father undermining their mother. This process was reported by the mothers, who noted how violent ex-partners sought to undermine them and their children. This was complicated when, as the practitioners reported, young males in single parent situations become the ‘man of the house’, perpetuating aggression against the mother. The young people were confused by the experience of stereotypical and gendered roles perpetuated by the perpetrator parent. Their experience of their maternal, non-abusing parent and their lack of learning or understanding about what constitutes appropriate youth-parent relationships, compounded the situation. Where this was met with a mother who felt intimidated and confused by what was happening, the situation could escalate into violence.

### Social media

Parents and practitioners perceived media and social/digital technology as having a negative impact upon young peoples’ behavior. Anecdotal information and popular rhetoric informed perspectives, as one of the practitioner participants noted “…social media has changed domestic abuse quite a lot …I’ve had someone sent to prison lately for Facebook things”. The rapid growth in technology was perceived by practitioners as providing a means through which to perpetrate abuse. Alternatively, digital media was also regarded as a positive and creative means to inform new, non-violent interventions for working with family dynamics. For example, the Respect program for young perpetrators uses a range of digitally facilitated interventions, which parents and young people found helpful.

### Coping tactics

The young people used a range of tactics, which could be described as either adaptive or deviant, to manage their situation. For example, Evie used a voodoo doll to help disperse her anger and tension. Following participation in the IDAS change program, she was able to positively channel her energies into other forms of activity, including martial arts. George displaced his anger, aiming it at his non-abusing mother. George was silenced by his father’s abuse and took out his rage and confusion on his mother. This was the only way he knew how to react to his entrapment with no one available to mediate his thoughts and behaviors. George had witnessed his mother being beaten on numerous occasions. When his parents had split up, he continued access visits with his father. Subjected to regular physical and psychological abuse, meted out by his alcoholic father and the father’s new partner, George was often locked in the house while the father and partner went out to get drunk. On one occasion, George was made to eat raw meat. On another he witnessed his father throw the partner downstairs. George did not know how to cope and the situation was finally brought to a head, and to the attention of statutory authorities, when his father attempted to abduct him.

The tension between the non-aggressor parent’s knowledge of ongoing abuse against their child, and that child’s capacity to challenge the abuse, or to disclose it to the non-abusing parent, appeared to be a complex dynamic that challenges the child-parent relationship. A parent may choose to deny or minimize the impact of the domestic abuse, for fear of the children being removed by social services; an invidious trap for the parent and for the child.

### Parental paralysis

Feeling trapped, immobilized, incompetent, lacking in confidence, alongside feeling low or depressed, could lead to ‘paralysis’ and inability to act to mitigate or mediate a situation of youth to parent aggression or violence. One mother captures the ambiguity and horror of the situation:

“…it frightened me, he was my son and I’d do anything for my children but I didn’t, I love, I love the bones of him but I didn’t like him at that time… and nobody [knew] you keep it in, you keep domestic violence in…”

The notion of parental paralysis was communicated by most practitioner respondents, who reported parents being unable to act in their own or their child’s best interests.

### Practitioners as wounded healers

The focus group elicited disclosures from several individuals who had experienced domestic violence as children. Each noted that they had not themselves gone on to become perpetrators; choosing instead to undertake preventative or ameliorative work through domestic violence services or organizations. Focus group and individual interview participants represented voluntary and statutory sector front line practitioners. Each conveyed the challenges, stresses and rewards of their role. Statutory sector workers experienced fear and hostility (from their client group) which impacted upon their perception of themselves in their work. As one person noted “…working with these sorts of people I go back to my own experiences as a child, of domestic violence. It occurred in my household; at the age of 5 I’d be lying in bed hearing [the violence]…”.

Perceived public distrust of statutory bodies exerted a negative impact. One practitioner noted “If I had a pound for every time a parent said to me ‘you’re going to take my child’, I’d be a millionaire”. Being constantly subjected to the complexities and challenges of working with domestic aggression and violence took its toll, impacting upon practitioner perceptions of humanity, as well as their capacity for self-care. One reported “…it’s a murky world when you work with people who commit sexual offences and domestic violence, it colors your view of society”. Another spoke of a case where a mother had described her child as evil and poignantly recounted:

“… no child is evil…when we came out the house, we were sat in the car and [co-worker] said to me ‘ I don’t think I can do this job anymore’ and I said ‘you can, we’ve just hit a little bit of a bad area, we will overcome this, but you’ve got to believe in yourself’”.

This scenario epitomized the psychological and emotional trauma experienced by some practitioners working with youth-to-parent domestic violence cases. Paradoxically, there was concurrent recognition of the positive and reparative impact of their work, including delivering interventions that positively changed young people’s lives.

### Bystanders

All of the practitioners referred to the way in which children and young people were captive bystanders in domestic violence contexts. They cannot escape and usually had heard or witnessed domestic violence, with no perceived exit or remission from the situation. George epitomized what some bystander children and young people are subjected to. George’s mother recalls how:

“… he had violent headaches, he wet the bed, he had nightmares, he made himself sick a couple of times after eating, which was a really big worry…withdrawn and smashing things up…”.

Although George’s violent father was separated from his mother, he still spent time at his father’s, groomed by the lure of treats. He frequently witnessed his father being violent with his new partner. Having already lived through his mother being treated violently, he was further traumatized and began behaving violently towards his mother. Over time, the mother became aware of what was happening with the father, whilst concurrently George became disillusioned with his father’s empty promises. Contact with his father ceased. The situation came to a head when the father attempted to abduct George, resulting in the police and social services being called in.

## Discussion

Few in-depth qualitative accounts of youth to parent domestic violence and abuse exist. This qualitative project offers three key findings that complement existing studies and contribute to the knowledge base on familial aggression and violence. Firstly, young people who are aggressive with their parent or caregiver are usually victims themselves, therefore it is inappropriate to pathologize them as ‘perpetrators’. Secondly, in the face of an aggressive or violent child, parents are far less resilient or competent than might be imagined. Here, the role of relational and communication skills programs – such as that developed in the UK by Respect and delivered by DVA agencies – are important. Thirdly, frontline practitioners working with children and adults exposed to domestic abuse and violence can experience vicarious trauma, as indicated by researchers in the field of trauma (Dagan et al. 2015; Killian et al. 2017; Newell et al. 2016. The self-care, wellbeing and support of frontline workers is not sufficiently foregrounded in the DVA literature, highlighting the need for further research into both the negative and positive effects for frontline staff such as counselors, therapists, social workers, youth case workers and police (Killian et al. 2017).

All three key findings relate to individuals’ capacities for resilience and agency, their relational abilities, and their mental health and emotional wellbeing. The significance of learning how to be with self and with others, across different types of relationships, featured across all three participant groups. Equally, the value of a multi-modal and relational approach to viewing DVA was indicated. Young people, parents, and practitioners, conveyed the multiple challenges of dealing with DVA and the ensuing powerful and paradoxical emotions and beliefs. As Abrahams (2010) notes, young people in situations of familial violence “…inhabit an unsafe and unpredictable world, with feelings and experiences that can be hard to process at any age.” (p.128). Certainly, the young participants in this project conveyed confused and conflicted perceptions and behaviors, including confusion associated with gender and age, supporting the current trends in research (Milaniak and Widon 2015). Additionally, children and young peoples’ resilience in the face of distressing and violent circumstances (Ehrensaft et al. 2003) warrants further research and we need in-depth case studies of the relational dynamics in youth to parent aggression and violence (Katz 2015) to complement large scale and quantitative studies.

### Who is the victim, who is the perpetrator?

The present project elicited the important question *who is the victim and who is the perpetrator?* The young participants had witnessed parental violence and their experiences and perceptions supported the notion that experiencing DVA can influence subsequent aggressive behavior (Calvete et al., 2015; Routt and Anderson 2011). Nevertheless, children and young people caught up in domestic violence situations are unwilling bystanders and victims. Children can become ‘forgotten people’ (Abrahams 2010, p.131) and demonizing the young person undermines opportunities to foster positive behavioral change and prevent future perpetration. Children who have experienced DVA have likely experienced developmental and relational trauma (see Rothschild 2017; Herman 1992). Trauma literature emphasizes the body’s response to traumatic experiences. The pathology created by positioning the child as purely ‘perpetrator’ or ‘victim’ is problematic as it overlooks the body’s response to trauma and the relational contexts of children and young people. It is therefore crucial to avoid over-simplifying the child as a ‘victim’ or ‘perpetrator’ and to recognize that young peoples’ relational positions are more complex. Although the psychological effects of children experiencing domestic violence are widely noted (Rothschild 2017; Herman 1992) their relational ways of coping remains an under-researched area. Victim and perpetrator constructs could perpetuate the ‘dysfunctional’ labelling of the child, therefore overlooking their lived and relational experiences (Burman 2017) and limiting the possibility for positive change.

Practitioners in the present study noted of the children they worked with “…they are not devils…” and “…no child is evil”. A child who phoned the police in an attempt to stop the violence demonstrated agency and resilience in the face of trauma (Killian et al. 2017); qualities that need to be developed in children and young people, as well as in those parents struggling in domestic violence situations.

### Parental resilience and competence

Youth to parent aggression and violence opposes the nature of an intimate relationship, such as a husband and wife, where one member of the relationship has the choice to leave and terminate a destructive relationship. On the contrary, a parent to youth relationship is limited in this respect. Parental responsibilities bring the potentiality for legal sanctions should the parent fail in their parenting duties. This could result in a parent feeling helpless, powerless and unable to disclose or act in cases of youth to parent abuse.

The psychological impact of domestic abuse on parents was significant and compounded earlier partner or childhood abuse; further eroding personal confidence and competence. Prior to disclosure of youth to parent DVA, parents experienced guilt, shame, fear, powerlessness, lack of support, and lack of awareness of who to turn to, mirroring findings in the literature (Holt 2011; McKenna et al. 2010; Sanderson 2008). The mothers conveyed gratitude for having access to a supportive professional program which elicited positive and productive ‘parenting tools’, including how to better manage relational conflict and boundaries in the parent-young person relationship.

Interventions for youth to parent DVA currently in use in the UK (see for example, Respect http://respect.uk.net) involve parents in attempts to tackle CPV, and place emphasis on working with parental guilt, shame, self-care, and boundaries. Restorative programs place emphasis on working with parents to minimize their ‘shame cycle’ and feelings of helplessness, and to promote ‘healthy relationships’ in the family system. Ideally, agencies can offer sanctuary to both parents and children who are affected by familial violence, rather than have interventions delivered by statutory agencies; their involvement is needed when there are no other options. Parenting tool kits and supportive strategies would seem essential (Hillberg 2011) to supporting early intervention and prevention, as well as to alleviating active abusive and violent behavior. Moreover, positive and reparative interventions which focus upon the parent-child relationship and the social construction of gender within familial contexts could inform productive and sustainable change (Wilcox 2012). Whilst cultural variations influence definitions of ‘healthy relationship’, a capacity to form productive and mutually respectful and rewarding relationships is, arguably, central to good mental health. Interventions need to be relevant to young people, parents and practitioners (Howarth et al. 2015) and serve to sever negative generational transmissions. Importantly, they need to be survivor-defined (Goodman et al. 2014), ensuring that those exposed to DVA inform development of interventions and services.

### Impact upon the practitioner

Practitioners felt overstretched and vulnerable; especially when sole working with DVA cases. Challenges were compounded by a perceived lack of appropriate work-based support. Arguably, practitioner wellbeing and self-care should be a core consideration across agencies dealing with domestic and interpersonal abuse and violence. Furthermore, a lack of self-care could contribute to practitioner traumatization (Newell et al. 2016) suggesting the need for further research on the impact of working with trauma. The *wounded healer* concept is recognized within Jungian psychological theories (Sedgwick 1994). Used in relation to a practitioner, it denotes someone who has experienced trauma or harm in their own past – as was the case with several of the practitioner participants. Key to dealing with past trauma is practitioner self-care and self-compassion (Sedgwick 1994). Interestingly, recent research indicates positive effects can accrue through working with vulnerable populations, including vicarious resilience and the development of self-care tactics (Killian et al. 2017; Newell et al. 2016). However further research is necessary in order to identify key factors operating in such situations.

In considering youth to parent DVA, most of the practitioners expressed concerns about the impact of social media upon young peoples’ behavior. However, research on the effect of web-based materials on abuse and violence is limited and inconclusive. Recent studies on the influence of social media on aggressive behavior indicate the importance of taking a rigorous and multi-dimensional approach to the issue (Diamond 2009; Phipps et al. 2017).

### Conclusion

The project was an in-depth, small-scale qualitative study therefore findings cannot be generalized to wider populations. We need to extend and deepen our understanding of the relational dynamics of youth to parent aggression and violence and the specific relationship contexts in which they occur. In particular, the parent-child relationship and the complex terrain of self-agency, resilience, relational power and coping tactics in the face of complex familial violence, require further investigation. Further research investigating the youth-parent relationship is essential, as well as ongoing development and evaluation of interventions to innovate relational resilience and coping strategies for both young people and parents. Developing a more sophisticated understanding of youth to parent violence and abuse, whilst evolving a better understanding of prevailing social, political and cultural influences, is essential.
